# Buccal mucosal graft urethroplasty in pan-urethral stricture in an adult patient with epidermolysis bullosa: a case report and review of literature

**DOI:** 10.1093/jscr/rjaf1082

**Published:** 2026-01-16

**Authors:** Huda Meshikhes, Dania Alseini, Hossam S El-Tholoth, Tala Al-Afraa

**Affiliations:** Urology Department, King Fahad Specialist Hospital, Ammar Bin Thabit St, Al Merikbat neighborhood, Dammam, 32253, Saudi Arabia; Urology Department, Prince Sultan Military Medical City, Makkah Al Mukarramah Rd, As Sulimaniyah, Riyadh, 12233, Saudi Arabia; Urology Department, Prince Sultan Military Medical City, Makkah Al Mukarramah Rd, As Sulimaniyah, Riyadh, 12233, Saudi Arabia; Urology Department, Prince Sultan Military Medical City, Makkah Al Mukarramah Rd, As Sulimaniyah, Riyadh, 12233, Saudi Arabia

**Keywords:** pan-urethral stricture, epidermolysis bullosa, buccal mucosal graft, urethroplasty

## Abstract

Epidermolysis bullosa is a rare genetic disorder that leads to skin fragility and the formation of blisters from the slightest trauma. There are 4 major types that have been described, with junctional and dystrophic types being more prone to result in genitourinary tract complications. The most common complication is meatal stenosis. Others include urethral stricture, urinary retention, ureteral stricture, bladder hypertrophy, and urinary tract infections. We report the successful outcome of urethroplasty using buccal graft mucosa for a 34-year-old male with epidermolysis bullosa who suffered from pan-urethral stricture.

## Introduction

Epidermolysis bullosa (EB) is a rare genetic disorder characterized by fragile skin and mucous membranes that blister, erode, and ulcerate with minimal trauma. This results from structural defects at the dermo-epidermal junction which reduce the resistance to mechanical stress. EB is classified into four types based on the location of these defects (ordered from most common): EB simplex (autosomal dominant), dystrophic EB (autosomal dominant or recessive), junctional EB (autosomal recessive), and Kindler EB (autosomal recessive). The overall incidence and prevalence of inherited EB were 19.6 and 8.2 per 1 million live births, respectively. This difference reflects significant morbidity and mortality, especially in severe forms [1]. The course of the disease initially presents at birth or early childhood with complaints of blistering, skin fragility, and non-healing wounds after trauma. EB may also involve organs beyond the skin, including—but not limited to—the eyes leading to corneal scarring or blindness, gastrointestinal tract with complications like esophageal strictures, pyloric atresia, gastroesophageal reflux, or constipation, musculoskeletal system manifesting as muscular dystrophy, pseudosyndactyly, or gait abnormalities, and the genitourinary tract. Consequently, a multidisciplinary team is paramount in EB management [2].

Pan-urethral stricture, involving the full urethral length, accounts for only 4.9% of all strictures. The condition may have an idiopathic cause or be brought on by trauma, inflammation, endoscopic urethral manipulations, or prior urethral surgery [3]. The most common inflammatory cause for pan-urethral stricture is lichen sclerosis, which accounts for 48.6% [4]. Although data on EB’s genitourinary involvement are limited, the National EB Registry from 1986 to 2022 reports complications prevalence reaching 31.1% in recessive dystrophic EB, 30.2% in junctional EB, and 16.6% in EB simplex. Meatal stenosis was the most common complication. Ongoing blistering and scarring can result in stricture formation. Other manifestations include urinary retention, bladder hypertrophy, hydronephrosis secondary to ureteral strictures, pyelonephritis, and cystitis [5].

This case report delves into the successful application of buccal graft mucosa (BMG) urethroplasty in an EB patient with pan-urethral stricture.

## Case presentation

A 34-year-old male known case of EB simplex, chronic kidney disease due to reflux nephropathy, and pan-urethral stricture. He underwent numerous interventions during childhood, including meatotomy, cystoscopies, and urethral dilatation. A Mitrofanoff was created in 2003 as he was unable to use the urethra for clean intermittent catheterization (CIC).

**Figure 1:**
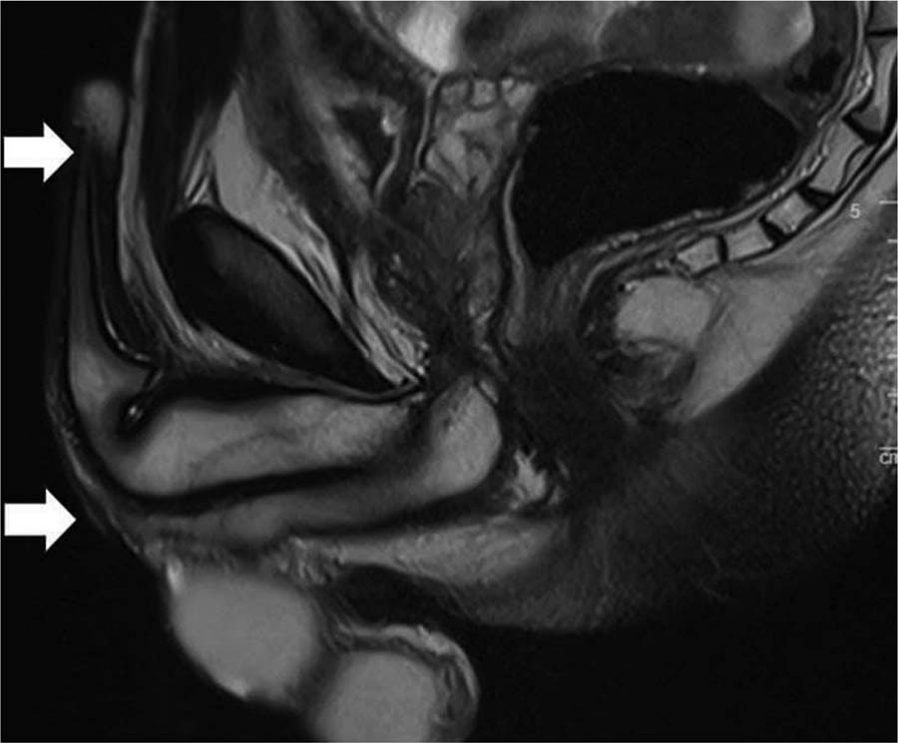
Sagittal MRI of the pelvis demonstrating a pan-urethral stricture. The urethra shows a long segment narrowing extending between the two arrows.

Physical examination revealed a soft and lax abdomen with a non-palpable bladder, and the urethral meatus was normally positioned without stenosis or blisters. A trial of urethrogram showed a pin-hole long segment urethra, limiting the quality of the study. Subsequently, a pelvic magnetic resonance imaging (MRI) was performed (Fig. 1), demonstrating attenuation of the prostatic urethral lumen with a low signal periurethral zone and faint high T2 signal in the membranous, bulbous, and penile urethra, supporting the diagnosis of pan-urethral stricture.

In June 2018, he underwent BMG urethroplasty. A 6 × 2 cm graft was harvested from both inner cheeks and applied dorsally using a Kulkarni one-stage pan-urethral approach. Immediately postoperatively, he developed multiple facial skin blisters, but the harvested graft site showed no signs of infection or ulcers. This event appeared to be incidental but disease-related and was most likely triggered by pressure from the face mask or adhesive contact from securing the endotracheal tube during anesthesia.

**Figure 2:**
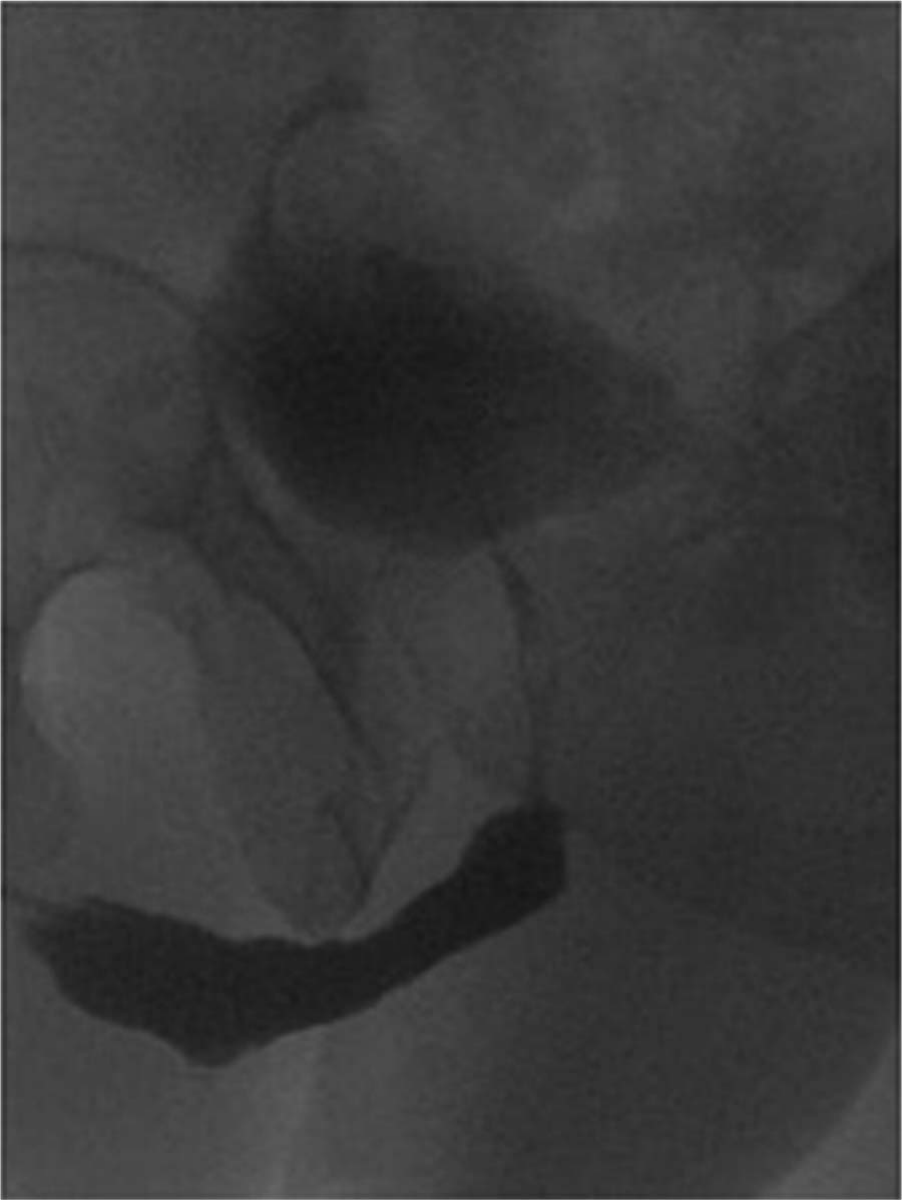
Post buccal graft mucosal urethroplasty urethrogram, showing wide urethra at the site of repair without recurrence.

The patient was able to void freely through the urethra and was kept on CIC to ensure low post-void residual volume, which led to the successful surgical closure of the Mitrofanoff stoma. At five-year follow-up, there was no evidence of recurrence of his disease, and his course was satisfactory. CIC was discontinued entirely over the last 2 years. His renal function remained stable with minimal post-void residuals. Cystourethroscopy confirmed a wide, healthy urethra at the graft site, without recurrence, blistering or ulceration. Follow-up urethrogram confirmed a patent repair without evidence of leak or stricture recurrence, and contrast reaching easily to the bladder (Fig. 2).

## Discussion

During our review of literature, we found only 3 adult cases of EB involving the genitourinary tract [6–8]. The first genitourinary manifestation was reported in a 3-year-old boy by Kretkowski in 1973 as a case of meatal stenosis [9]. The most frequent genitourinary complication was meatal stenosis, occurring in 11.6% and 8% of junctional and recessive dystrophic EB, respectively [5]. Unlike the reported cases, 1 case presented initially with genitourinary complications of EB, including meatal stenosis with peri-meatal blistering and difficult voiding in a 6-year-old boy. He was treated as a case of neurogenic bladder with oxybutynin and CIC. Later, he developed urinary retention and multiple erosions in different areas of his body, where a diagnosis of junctional EB was confirmed by a skin biopsy of the lesion, and subsequent definitive continent cutaneous urinary diversion with Mitrofanoff was created [10].

To our knowledge, our case is the only case that performed BMG urethroplasty in a patient with EB and showed a satisfactory result without recurrence. The first case that reported urethral stricture in junctional EB was in 1987 [6]. To date, only 3 cases of uretheral stricture secondary to EB were found [7, 8, 11]. One patient developed pan-urethral stricture and was only managed by cystostomy and CIC [7]. Another study described a patient with junctional EB having a cavernous urethral stricture and underwent multiple urethroplasties, including lingual graft and penile skin flap, that both failed and ended with perineal urethrostomy [8].

There are many reconstructive options for pan-urethral strictures. BMG was first introduced in 1894; since then, it has become more popular worldwide with a success rate of 83.7% in pan-urethral stricture disease [3]. BMG urethroplasty is not ideal in cases involving infectious diseases affecting the mouth, including candida, lichen sclerosis, varicella, or herpes virus [12]. However, there is no clear contraindication to use BMG in patients who are affected by a dermatological disease like EB, especially when not involving the mucosa, which makes it a valid option to use in such a situation, like in our case.

## Conclusion

This case demonstrates the success of using BMG for pan-urethral stricture despite the patient having EB, which may make the oral mucosa an option in such cases. Further studies are needed to confirm the possibility of using BMG in such patient population.
